# High SAD PERSONS scale scores are associated with subsequent psychiatric admission after suicide attempts: A single‐center retrospective study

**DOI:** 10.1002/pcn5.70342

**Published:** 2026-05-25

**Authors:** Tatsunori Nagamura, Takero Terayama, Kohei Yamada, Hiroyuki Toda, Tetsuro Kiyozumi

**Affiliations:** ^1^ Department of Traumatology and Critical Care Medicine National Defense Medical College Saitama Japan; ^2^ Department of Emergency and Critical Care Medicine Japan Self‐Defense Force Central Hospital Tokyo Japan; ^3^ Department of Psychiatry National Defense Medical College Saitama Japan

**Keywords:** emergency department, psychiatry, self‐harm, self‐injury, suicide attempt

## Abstract

**Aim:**

The SAD PERSONS scale (SPS) assesses the risk of future suicide attempts; however, its utility in patients with severe physical injuries is unclear. This study explored the association between high SPS scores and subsequent psychiatric admission (SPA) among patients transported to a tertiary emergency center following suicide attempts.

**Methods:**

This single‐center, retrospective observational study included patients transported to our tertiary emergency center between January 1, 2019, and December 31, 2023. Patients were enrolled based on their scores on the SPS: ≥5 points (High SPS group) or <5 points (Low SPS group). The primary outcome was SPA; the secondary outcomes were physical complications, length of stay in the emergency department, involuntary psychiatric admission, and re‐suicide attempts in the general ward.

**Results:**

Of 199 patients, 58 (29.1%) were in the High SPS group and 141 (70.9%) in the Low SPS group, with 67 SPA and 61 involuntary admissions. The High SPS group had significantly higher SPA than the Low SPS group (67.2% vs. 19.9%, *p* < 0.01). Physical complications, length of stay in the emergency department, and involuntary psychiatric admissions were significantly higher in the High SPS group than in the Low SPS group. Re‐suicide attempts in the general ward did not significantly increase. Multiple logistic regression analysis revealed that an SPS score of ≥5 points was significantly associated with SPA (odds ratio 6.97; 95% confidence interval, 2.85–17.1; *p* < 0.01).

**Conclusion:**

High SPS scores are associated with SPA. This finding helps emergency physicians predict psychiatric consultations and admission.

## Graphical abstract

In this single‐center retrospective study (2019–2023), we evaluated whether the SAD PERSONS Scale (SPS) predicts subsequent psychiatric admission (SPA) in patients presenting with self‐harm. Patients with SPS ≥ 5 had a significantly higher rate of SPA than those with lower scores (67.2% vs. 19.9%), and SPS ≥ 5 was independently associated with SPA (adjusted odds ratio [OR] 6.97; 95% confidence interval [CI], 2.85–17.1). These findings suggest that SPS may be a practical tool for early identification of patients requiring psychiatric admission in emergency settings.

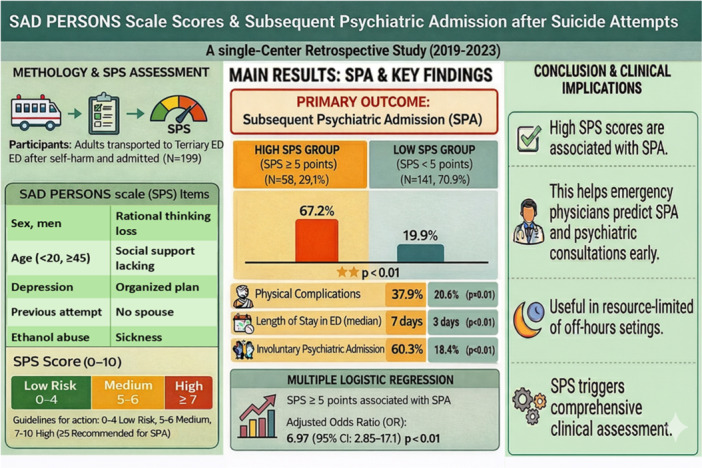

## INTRODUCTION

Suicide is a serious public health problem, resulting in more than 20,000 annual deaths in Japan, and is the leading cause of death among 15–39‐year‐olds.^1^, ^2^ As more than 30,000 patients have been transported to the emergency department (ED) because of self‐harm,^3^ emergency physicians need to provide appropriate initial care for suicide, even in hospitals without full‐time psychiatrists. Patients transported to the ED because of suicide attempts often require subsequent psychiatric treatment in psychiatric wards (subsequent psychiatric admission [SPA]) after physical treatment. Thus, early prediction of SPA by non‐psychiatrists is required for better clinical outcomes in these patients.

The SAD PERSONS scale (SPS) is a simple scale used by non‐psychiatrists to assess the risk of future suicide attempts.^4^ However, the SPS has only been validated in a limited population. Okamoto et al. compared SPS scores between SPA and non‐SPA groups among patients transported to the ED because of suicide attempts in a secondary emergency hospital and reported that total SPS scores were associated with SPA.^5^ However, it is unclear whether the SPS is also useful in more physically severe patients, such as those transported to tertiary emergency centers.

This study aimed to assess whether high SPS scores are associated with SPA in patients transported to a tertiary emergency center for suicide attempts.

## METHODS

### Study design, setting, and participants

This single‐center, retrospective, observational study was approved by the Ethics Committee of National Defense Medical College (approval ID: 5030). Patients transported to the ED at National Defense Medical College Hospital between January 1, 2019, and December 31, 2023, were enrolled.

The inclusion criteria were as follows: (1) age ≥ 18 years, (2) transported because of any form of self‐injury, and (3) required admission for physical treatment. The exclusion criteria were as follows: (1) cardiopulmonary arrest on arrival, (2) accidental injury after further evaluation post‐admission, (3) death before a psychiatric consultation, (4) refusal to receive medical treatment, (5) admission for respite care, and (6) discharge within 24 h after admission. Participants were classified into SPS scores ≥ 5 (High SPS group) or <5 (Low SPS group).

### SPS

**Figure 1:**
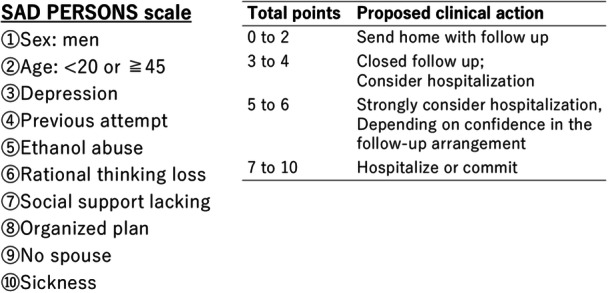
The SAD PERSONS scale and guidelines for action with the scale. The SAD PERSONS scale consists of risk factors for suicide attempts, with each factor scored as one point if applicable, resulting in a total score ranging from 0 to 10.

The SPS consists of 10 risk factors for predicting future suicide reattempts (Figure 1).^4^ Each item is scored as one point if applicable, resulting in a total score ranging from 0 to 10. The risk level is evaluated as follows: 0–4 points, low risk; 5–6 points, medium risk; and 7–10 points, high risk. In general, patients with an SPS score of ≥5 points are strongly recommended admission for psychiatric care.^4^ Some prior studies have reported low sensitivity (1%–49%) and positive predictive accuracy (5.3%–25%) of SPS for predicting future suicide attempts.^6^, ^7^ However, each item of the SPS is often considered crucial for managing suicide attempts; thus, the SPS is widely used for evaluation.

### Mental health system for psychiatric emergency in Japan

Patients with severe psychiatric states are often unable to provide informed consent for treatment because of the nature of their illnesses, even when medical care is indicated. In such cases, involuntary hospitalization can be initiated if Designated Physicians of Mental Health consider admission and prompt care necessary for their health in Japan.

In Japan, the Act on Mental Health and Welfare of the Mentally Disabled was enacted in 1988. In addition, a system of Designated Physicians of Mental Health was established to recognize the national qualifications of doctors who exclusively make decisions regarding involuntary admission in psychiatric treatment.^8^ Designated Physicians of Mental Health examine patients based on the criteria set out by the law.

### Management of patients with suicidal attempts in our hospital

National Defense Medical College Hospital is a tertiary emergency center with full‐time psychiatrists, Designated Physicians of Mental Health, and psychiatric inpatient beds. However, we do not have a high‐acuity psychiatric emergency ward specifically designed for the intensive care of patients with acute or severe psychiatric conditions.

The Psychiatry Department operates an emergency liaison team that monitors all patients admitted to the emergency ward. Joint conferences between psychiatrists and emergency physicians are regularly held to determine treatment plans.

### Data collection

Data on the patients' age, sex, past psychiatric medical history, methods of suicide attempts, psychiatric diagnosis, SPS score at admission, suicide reattempts in general wards, length of stay in the ED, and physical complications were collected from electronic medical records. “Poisoning” included an overdose of liquid medicine or carbon monoxide poisoning. “Stab wounds” included cuts and wounds on the trunk and neck. “Accidental injury” was defined as cases that were not considered suicide attempts after a psychiatric evaluation by a psychiatrist. “Respite care” in this study refers to short‐term psychiatric admissions intended to provide temporary support for patients in order to relieve caregiver burden. These cases were identified based on explicit documentation in the admission record. Several SPS items were operationally defined in accordance with prior research.^5^ “Social support lacking” was defined as significant psychosocial stressors or isolation, such as poor family relationships, unemployment, decline in social status, financial loss, bereavement, domestic violence, or unstable engagement in ongoing medical care. “Organized plan” was defined as evidence of structured or highly lethal suicidal intent, including the use of high‐lethality methods (e.g., hanging, jumping from height, gas inhalation, or stab wounds), the combination of multiple methods, or elaborate preparation (e.g., writing a suicide note). “Sickness” was defined as chronic debilitating medical conditions causing substantial functional impairment or severe distress, including incurable illnesses (e.g., malignant tumors or neurodegenerative diseases), marked decline in activities of daily living after trauma or stroke, or treatment—resistant chronic pain disorders. “Rational thinking loss” was defined as the presence of psychotic symptoms (e.g., hallucinations or delusions), delirium, severe cognitive impairment, or marked disorganization of thought documented in the medical records.

In this study, the SPS score was assessed retrospectively by the first author (T.N.), who is an emergency physician. Subjective items were coded as positive only when explicitly documented and otherwise as negative; thus, no variables were treated as missing. This approach was chosen because the proportion of undocumented elements was too large to allow reliable imputation. The SPS score was primarily calculated based on medical records documented before the initial psychiatric consultation. However, when sufficient information from the patient or family was not available at that time, the score was supplemented using information recorded in the psychiatrist's first assessment, typically documented within the first week of admission.

### Outcomes

The primary outcome was SPA. Secondary outcomes were any physical complications, length of stay in the ED, involuntary psychiatric admission, and repeated suicide attempts in the general wards. Physical complications were defined as those requiring the following treatments: tracheal intubation for consciousness disturbance or aspiration pneumonia, any surgery under general anesthesia, hemodialysis for toxins or hyperkalemia, and prosthetics for fracture of the limbs or spine.

### Statistical analysis

Data are presented as means and standard deviations, numbers and percentages, or medians and interquartile ranges. Continuous variables with normal distribution were compared using Student's *t‐*test. Categorical variables and continuous variables without normal distribution were compared using the *χ* ^2^ test, Fisher's exact test, or the Mann–Whitney *U* test, respectively. The association between a high SPS score and SPA was evaluated using multivariable logistic regression analysis and expressed as odds ratios (ORs) with 95% confidence intervals (CIs). The model was adjusted for psychiatric diseases (schizophrenia, mood disorders, and borderline personality disorder), which often involve self‐harm, as well as additional clinically relevant baseline variables that attending clinicians judged to be important potential confounders, such as age, sex, and method of suicide attempt (overdose and stab wounds).

All statistical analyses were performed using EZR software (version 2.4.2; Japan). Statistical significance was defined as a two‐sided *p* < 0.05.

## RESULTS

### Patients and baseline characteristics

**Figure 2:**
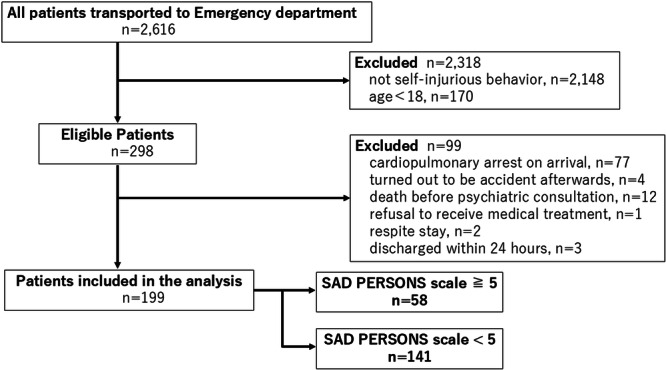
Flow diagram of the study participant selection process. A total of 2616 patients met the selection criteria. Through the inclusion and exclusion criteria, patients were divided into two groups based on their scores on the SAD PERSONS scale: ≥5 points (*n* = 58) and <5 points (*n* = 141).

A total of 199 patients met the inclusion criteria. Of these, 58 (29.1%) and 141 (70.9%) were enrolled in the High SPS and Low SPS groups, respectively (Figure 2). The number of SPA and involuntary admissions was 67 (33.6%) and 61 (30.6%), respectively.

At baseline, age (47 [39–57] vs. 32 [25–48] years, *p* < 0.01), male sex (67.2% vs. 23.4%, *p* < 0.01), and history of schizophrenia (20.7% vs. 8.5%, *p* = 0.029) were significantly higher in the High SPS group than in the Low SPS group (Table 1). Overdose was significantly higher in the Low SPS group than in the High SPS group (34.5% vs. 69.5%, *p* < 0.01), and the incidence of stab wounds was significantly higher in the High SPS group than in the Low SPS group (24.1% vs. 4.3%, *p* < 0.01) (Table 1).

**Table 1:** Baseline characteristics of the included patients.

	SPS ≥ 5 (*n* = 58)	SPS < 5 (*n* = 141)	*p*‐value
Age, years	47 [IQR, 39–57]	32 [25–48]	<0.01
Sex, male	39 (67.2%)	33 (23.4%)	<0.01
*Past psychiatric medical history*			
Dementia	2 (3.4%)	10 (7.1%)	0.50
Alcoholic	2 (3.4%)	2 (1.4%)	0.58
Schizophrenia	12 (20.7%)	12 (8.5%)	0.029
Bipolar affective disorder	5 (8.6%)	24 (17.0%)	0.18
Depressive disorder	13 (22.4%)	35 (24.8%)	0.86
Generalized anxiety	0 (0%)	4 (2.8%)	0.32
Panic disorder	0 (0%)	5 (3.5%)	0.32
Obsessive‐compulsive disorder	0 (0%)	1 (0.7%)	1
Adjustment disorder	0 (0%)	2 (1.4%)	1
Dissociative disorder	1 (1.7%)	6 (4.3%)	0.68
Borderline personality disorder	0 (0%)	7 (5.0%)	0.11
Developmental disorder	3 (5.2%)	3 (2.1%)	0.36
Disturbance of activity and attention	1 (1.7%)	5 (3.5%)	0.67
Other psychiatric disorder	2 (1.7%)	9 (5.7%)	0.52
No diagnosis	20 (34.5%)	43 (30.5%)	0.62
*Methods of suicide*			
Overdose	20 (34.5%)	98 (69.5%)	<0.01
Poisoning	10 (17.2%)	13 (9.2%)	0.14
Stab wound	14 (24.1%)	6 (4.3%)	<0.01
Self‐inflicted wrist cutting	3 (5.2%)	8 (5.7%)	1
Hanging	4 (6.9%)	11 (7.8%)	1
Falling from height	11 (17.2%)	14 (10.4%)	0.10
Railway suicide	1 (1.7%)	0 (0%)	0.29
Self‐immolation	1 (1.6%)	1 (0.7%)	0.50

Abbreviations: IQR, interquartile range; SPS, SAD PERSONS scale.

### SPA and association between high SPS scores and SPA

The median SPS was 5 points in the High SPS group and 3 points in the Low SPS group. SPA was significantly higher in the High SPS group than in the Low SPS group (67.2% vs. 19.9%, *p* < 0.01) (Table 2). Multiple logistic regression analysis showed that an SPS score of ≥5 was significantly associated with SPA after adjusting for all clinically relevant covariates (adjusted OR 6.97; 95% CI, 2.85–17.1; *p* < 0.01) (Table 3).

**Table 2:** Comparison of outcomes between groups.

	SPS ≥ 5 (*n* = 58)	SPS < 5 (*n* = 141)	*p*‐value
SPS	5.0 [IQR, 5–6]	3.0 [2–4]	<0.01
Subsequent psychiatric admission, *n* (%)	39 (67.2%)	28 (19.9%)	<0.01
Involuntary hospitalization	35 (60.3%)	26 (18.4%)	<0.01
Any physical complications, *n* (%)	22 (37.9%)	29 (20.6%)	0.01
Length of stay in the emergency department, days	7 [3–13.7]	3 [2–8]	<0.01
Repeated suicide attempts in general wards	0 (0%)	0 (0%)	1

Abbreviations: IQR, interquartile range; SPS, SAD PERSONS scale.

**Table 3:** Multivariable logistic regression analysis of SAD PERSONS scale (SPS) scores for subsequent psychiatric admission.

	OR	95% CI
SPS score ≥ 5	6.97	2.85–17.1
Past history of schizophrenia	2.11	0.75–5.91
Past history of mood disorder	0.72	0.34–1.50
Past history of borderline personality disorder	0.68	0.078–6.02
Age	1.03	1.01–1.05
Sex, male	0.44	0.18–1.08
Methods of suicide, overdose	0.39	0.18–0.85
Methods of suicide, stab wound	2.46	0.72–8.4

Abbreviations: OR, odds ratio; CI, confidence interval; SPS, SAD PERSONS scale; mood disorder, depressive disorder and bipolar affective disorder. OR was adjusted for schizophrenia, mood disorder, borderline personality disorder, age, sex, overdose, and stab wounds.

### Secondary outcomes

Physical complications (37.9% vs. 20.6%, *p* = 0.01), length of stay in the ED (7 [3–13.7] vs. 3 [2–8] days, *p* < 0.01), and involuntary psychiatric admission (60.3% vs. 18.4%, *p* < 0.01) were significantly higher in the High SPS group than in the Low SPS group (Table 2). No significant difference was observed in the proportion of repeated suicide attempts during general ward hospitalization (0% in both groups, *p* = 1.00; Table 2).

## DISCUSSION

This study revealed that a score of ≥5 points on the SPS was associated with SPA among patients transported to a tertiary emergency center owing to suicide attempts. Our findings contribute to the appropriate management of suicide attempts in EDs at tertiary emergency centers and lead to the early initiation of psychiatric treatment.

Three previous studies have investigated the association between SPS and SPA; however, their results were inconsistent (Table 4). Saunders et al. reported that among 126 patients transported to a major general hospital because of suicide attempts, 5 (3.9%) required SPA and that a score of ≥7 points on the SPS was not associated with SPA.^9^ In contrast, Chang et al. and Okamoto et al. demonstrated a significant association between high SPS and SPA, although these two studies had different SPS cutoff points. These differences may be due to variations in patient backgrounds and healthcare systems.^5^, ^10^

**Table 4:** Summary of backgrounds in related studies.

	Chang BP, 2015	Saunders K, 2014	Okamoto, 2020	Nagamura, 2026
Study design	Prospective	Prospective	Retrospective	Retrospective
Study site	United States of America	United Kingdom	Japan	Japan
Clinical setting				
Type of hospital	University affiliated teaching hospital	Major general hospital	Secondary emergency hospital	Tertiary emergency hospital
Full‐time psychiatrists	Unknown	No	Yes	Yes
Psychiatric wards	Yes	No	Yes	Yes
Sample size (*n*)	50	126	143	199
Inclusion criteria	(1) Age ≥ 18 years (2) Chief complaint of “suicide ideation,” “thinking of hurting myself,” “I want to die,” or “SI”	All self‐harm patients: (1) Any act of self‐poisoning or self‐injury (2) Irrespective of the apparent purpose of the act, including degree of suicide intent	All patients with the possibility of self‐harm	(1) Age ≥ 18 years (2) Transported owing to any form of self‐injury (3) Required admission in general wards for some physical treatments
Age, years	36.4 (range: 20–57)	33.0 ± 14.5	42 ± 17	42.1 ± 18.8
Sex, female, *n* (%)	28 (56.0%)	72 (57.1%)	112 (78%)	127 (63.8%)
Suicide method	Suicidal ideation (excluding actual suicide attempt)	Overdose (81%) Self‐inflicted cutting (11%)	Overdose (70.6%) Hanging (9.1%) Self‐inflicted wrist cutting (8.4%) Jumping (7.7%) Poisoning (3.5%)	Overdose (59.2%) Jumping (12.6%) Poisoning (11.6%) Stab wound (10.1%) Hanging (7.5%) Self‐inflicted wrist cutting (5.5%)
SPS score	7.2 ± 1.6	3.8 ± 1.5	3.7 ± 1.6	3.4 ± 1.7
Association between high SPS score and SPA	Significant (cutoff point, 7)	Not significant (cutoff point, 7)	Significant	Significant (cutoff point, 5)
LOS in ED (SPA vs. non‐SPA, days)	Unknown	Unknown	Unknown	7 versus 3
SPA	22 (44.0%)	5 (3.9%)	47 (32.8%)	67 (33.6%)

Abbreviations: LOS in ED, length of stay in the emergency department; SPA, subsequent psychiatric admission; SPS, SAD PERSONS scale.

A score of ≥5 points on the SPS was associated with SPA in our study. However, the number of SPA in the study by Saunders et al. was much smaller than that in ours (5 vs. 67 cases). The number of psychiatric beds in the United Kingdom in 2020 was smaller than that in Japan (23,658 vs. 329,692) because of the national health policy of psychiatric deinstitutionalization.^11^ As the United Kingdom has made progress in closing or reducing large psychiatric hospitals and developing comprehensive community‐based mental health services for patients with severe mental illness,^12^ it is one of the countries with the lowest psychiatric admission rates. This may explain why the effectiveness of SPS was not demonstrated by Saunders et al. In the study by Chang et al., the SPS score is considerably higher than that in our study (7.2 vs. 3.4 points). All patients in their study had suicidal ideation, which contributes to a higher SPS score, as the scale is composed of 10 risk factors for suicide attempts. Okamoto et al.'s study was conducted in Japan; therefore, the same social background and medical system resulted in similar patient backgrounds and SPS scores as those in our study. However, the baseline characteristics were slightly different (Table 5). Their study included many patients with depression and previous suicide attempts, whereas our study included many patients with a loss of rational thinking and organized plans. The total scores for these factors canceled each other, resulting in similar SPS scores.

**Table 5:** Comparison of scores on the SAD PERSONS scale in Okamoto's study and our study.

SPS	Okamoto, 2020	Nagamura, 2026
Sex, male, *n* (%)	31 (22%)	72 (36.1%)
Age; <20 or >45, *n* (%)	73 (51.0%)	85 (42.7%)
Depression, *n* (%)	94 (64.7%)	58 (29.1%)
Previous attempt, *n* (%)	93 (65.0%)	82 (41.2%)
Ethanol abuse, *n* (%)	27 (18.8%)	21 (10.5%)
Rational thinking, *n* (%)	15 (10.4%)	40 (20.1%)
Social supports lacking, *n* (%)	69 (48.2%)	135 (67.8%)
Organized plan, *n* (%)	19 (13.2%)	78 (39.1%)
No spouse, *n* (%)	84 (58.7%)	100 (50.2%)
Sickness, *n* (%)	20 (13.9%)	12 (6.0%)
SPS score	3.7 ± 1.6	3.4 ± 1.7

Abbreviation: SPS, SAD PERSONS scale.

Our findings have practical implications for the acute management of patients who attempt suicide. In emergency settings, especially where full‐time psychiatric coverage is limited, the SPS can serve as a simple and rapid screening tool to identify individuals at high risk for SPA. By applying a cutoff of 5 points, emergency physicians may better anticipate the need for psychiatric consultation and admission early in the patient's clinical course. This early recognition is particularly valuable in resource‐limited environments or during off‐hours, when psychiatric evaluation may be delayed. In addition, since patients with high SPS scores also present with more physical complications and longer ED stays, early identification may support more comprehensive care planning, including physical and psychiatric interventions. Furthermore, given that involuntary admission was significantly more common in the High SPS group, early identification could also facilitate the legal and administrative preparations necessary under Japan's Mental Health and Welfare Law. Taken together, our findings support the clinical utility of the SPS not only as a suicide risk screening tool but also as a decision‐making aid for SPA in the acute phase. Importantly, the SPS is not intended to justify or replace structured psychiatric assessment; rather, its value lies in functioning as an early trigger that raises awareness among emergency physicians, facilitates timely decision‐making, and serves as an educational tool to improve the initial management of suicide attempts. Prospective validation and integration of local protocols may further improve patient care. However, in real‐world emergency practice, decisions regarding psychiatric admission are not determined solely by suicide risk scores. Physical recovery status after emergency treatment and the availability of reliable caregivers or social support systems are also important factors. Even patients with high SPS scores may occasionally be managed without psychiatric admission if their physical condition is stable and adequate social support is confirmed. Therefore, the SPS should be interpreted within the broader clinical context rather than used as an isolated determinant of admission decisions.

The SPS consists mainly of static and historical risk factors, which limits its ability to assess imminent suicide risk. Recent research emphasizes dynamic psychological constructs such as perceived burdensomeness and thwarted belongingness, as described in Joiner's Interpersonal Theory of Suicide.^13^ Our findings support the use of the SPS not as a stand‐alone predictive tool, but as a trigger for more comprehensive clinical assessment that includes dynamic psychosocial evaluation. Furthermore, decisions of psychiatric admission are influenced by multiple factors beyond the SPS score itself. Our objective was not to validate or modify the predictive performance of the SPS, but to examine its potential utility as an initial screening indicator in emergency settings where psychiatric consultation is not always immediately available. In real clinical practice, psychiatrists consider numerous contextual factors—including local medical culture, institutional systems, and available resources—when determining the need for psychiatric admission. We fully acknowledge that, from a psychiatric perspective, an SPS‐based approach has inherent limitations and cannot substitute for comprehensive specialist evaluation. Rather, it reflects the practical constraints of emergency medicine, where immediate psychiatric involvement is not always feasible, and highlights the need for improved pathways linking emergency and mental health services. Importantly, previous validation studies have reported that the SPS has limited sensitivity, potentially classifying a substantial proportion of high‐risk individuals as low risk, which may lead to an underestimation of subsequent self‐harm.^14^, ^15^ Furthermore, several reviews have cautioned that risk scales should not be used in isolation to determine patient management or to predict self‐harm outcomes.^6^, ^16^ Therefore, while the SPS may serve as a useful first step for non‐psychiatric clinicians, it is desirable that all patients with suicide attempts ultimately undergo a psychiatric evaluation to ensure appropriate disposition.

This study has certain limitations. First, our findings lacked external validity in regions with different emergency and psychiatric medical systems. In particular, the presence of a dedicated psychiatric emergency ward, the availability of full‐time psychiatrists, and national policies regarding the psychiatric healthcare concept are likely to have a substantial impact on these results. However, our findings remain important as they address a population in which the SPS has not been previously validated, thereby addressing gaps in the existing evidence. Second, some confounding factors were unadjusted for owing to the retrospective study design. Third, SPS scores might have been underestimated when patient medical records were insufficient. As described in the Methods section, subjective items were coded as positive only when explicitly documented and otherwise as negative in this study, which may have led to underestimation of SPS scores. Fourth, because the assessment was conducted by a single evaluator, inter‐rater reliability could not be calculated. Future prospective studies should evaluate the inter‐rater reliability of SPS scoring between emergency physicians and psychiatrists. Such investigations may further clarify the role of the SPS as a shared assessment tool bridging emergency medicine and psychiatric care.

In conclusion, among patients transported to a tertiary emergency center owing to suicide attempts, a score of ≥5 points on the SPS was associated with SPA. For patients with high SPS scores, careful efforts should be made to ensure appropriate post‐suicide care, including prompt psychiatric consultations.

## AUTHOR CONTRIBUTIONS

Tatsunori Nagamura, Takero Terayama, and Kohei Yamada designed the study, analyzed and interpreted the data, drafted the manuscript, and performed the statistical analysis. Hiroyuki Toda and Tetsuro Kiyozumi critically revised the manuscript for important intellectual content. All authors read and approved of the final manuscript.

## CONFLICT OF INTEREST STATEMENT

The authors declare no conflicts of interest.

## ETHICS APPROVAL STATEMENT

Approval of the research protocol: This study was approved by the Ethics Committee of the National Defense Medical College (approval ID: 5030).

## PATIENT CONSENT STATEMENT

We applied an opt‐out method on the institutional website to obtain patient consent.

## CLINICAL TRIAL REGISTRATION

N/A.

## Data Availability

The data that support the findings of this study are available from the corresponding author upon reasonable request.
